# Evaluation of the diagnostic accuracy of mammography and ultrasonography for breast cancer diagnosis using histopathology as the reference standard: a cross-sectional study

**DOI:** 10.1097/MS9.0000000000004369

**Published:** 2025-11-18

**Authors:** Sultan A. Alotaibi, Awadia Gareeballah, Jawwad Sami Ur Rahman, Wael A. Faqihi, Sara Ali, Meaad Elbashir, Nisreen Haj, Kawthar Mohammed Sharief Abdulrhman, Raga Ahmed Abouraida Ahmed, Ahmed Hazazi, Babar Ali

**Affiliations:** a Department of Radiological Sciences and Medical Imaging, College of Applied Medical Sciences, Majmaah University, Al Majmaah, Saudi Arabia; b Department of Diagnostic Radiology Technology, College of Applied Medical Sciences, Taibah University, Al-Madinah al-Munawwarah, Saudi Arabia; c Department of Biomedical Engineering, Riphah International University, Islamabad, Pakistan; d Department of Radiological Sciences, College of Applied Medical Sciences, Najran University, Najran, Saudi Arabia; e Diagnostic Radiography Technology Department, Faculty of Nursing and Health Sciences, Jazan University, Jazan, Saudi Arabia; f Department of Radiology, Alghad College for Applied Medical Science, Riyadh, Saudi Arabia; g Department of Radiological Sciences, College of Applied Medical Sciences, King Khalid University, Abha, Saudi Arabia; h Department of Public Health, Faculty of Health Science, Saudi Electronic University, Riyadh, Saudi Arabia‬‏‏; i Department of Research, Medical Research Circle, Bukva, DR Congo

**Keywords:** BI-RADS, breast cancer, diagnostic accuracy, histopathology, mammography, ultrasonography

## Abstract

**Background and aim:**

Breast cancer remains the most common malignancy and the leading cause of cancer-related death among women globally, especially in low- and middle-income countries. Early and accurate diagnosis is critical to improving survival outcomes. This study compares the diagnostic accuracy of ultrasonography and mammography in detecting breast carcinoma, using histopathology as the gold standard.

**Methods:**

A cross-sectional descriptive study was conducted in a radiology department at a tertiary care hospital, including 240 female patients presenting with clinically palpable breast lesions. Each participant underwent both mammography and ultrasonography prior to biopsy. Data collection included lesion characteristics, imaging findings, and histopathological results. Diagnostic performance was evaluated using sensitivity, specificity, positive predictive value (PPV), negative predictive value (NPV), and overall accuracy.

**Results:**

Ultrasound demonstrated a consistent diagnostic accuracy of 86.3% in both breasts, with high NPVs (≥95.7%) and good sensitivity in dense tissue. Mammography showed variability between sides: accuracy was 81.7% on the right and 94.6% on the left, with left-sided sensitivity reaching 100%. Ultrasound was more effective in identifying hyperechoic lesions and smooth margins, while mammography was superior in detecting calcifications and architectural distortions. Statistical testing confirmed significant associations (*P* < 0.001) for all modality comparisons with histopathology.

**Conclusion:**

This study highlights the complementary diagnostic value of ultrasonography and mammography in the evaluation of breast carcinoma. Ultrasound demonstrated consistent diagnostic accuracy and strong NPV, making it particularly valuable in premenopausal women and those with dense breast tissue. Mammography, on the other hand, showed superiority in detecting microcalcifications and subtle architectural distortions, underscoring its indispensable role in identifying ductal carcinoma *in situ* and early-stage malignancies.

## Introduction

Breast cancer continues to be the most common malignancy and the leading cause of cancer-specific mortality in females worldwide^[1,2]^. Based on the GLOBOCAN 2022 data published by the International Agency for Research on Cancer (IARC), there were about 2.3 million new breast cancer diagnoses in the world in 2020, accounting for 11.7% of all cancer diagnoses, and responsible for 685 000 deaths each year^[3]^. It overtook lung cancer as the most frequently diagnosed cancer in females^[4]^. Additionally, the lifetime risk of breast cancer development has been estimated to be one in eight females worldwide, with incidence rising in areas hitherto classified as low-risk. The prevalence of breast cancer is not evenly distributed. Higher inciaccountingdence rates – over 80 per 100 000 women – are seen in the developed countries owing to lifestyle factors and good screening programs, while lower incidence but relatively higher mortality in the case of the low- and middle-income countries (LMICs) results mainly from late presentation, lack of awareness, and inadequate access to diagnostic and therapeutic facilities^[5–7]^. In South Asia, particularly Pakistan, breast cancer is a major public health concern. Pakistan has the highest breast cancer incidence rate in Asia, with over 90 000 new diagnoses and 40 000 deaths each year. One in every nine Pakistani women is at risk of developing breast cancer in her entire lifetime^[8–12]^.

Early identification is key to enhancing survival, with 5-year survival of over 85% when the disease is diagnosed at the early stage. When the cancer reaches the late stage; however, the figure falls to below 25%^[13,14]^. Mammography continues to be the preferred gold standard for cancer of the breast owing to its capability to identify early tumors and microcalcifications not amenable to clinical examination. Its contribution to the prevention of breast cancer mortality has been widely demonstrated, especially in women aged between 50 and 69 years, in whom it reduces mortality by 20–30%^[14–16]^.

Mammography, though, has limitations, particularly in dense breasts and in young women, in whom its sensitivity falls below 50%. Dense fibroglandular breast not only obscures small tumors but also qualifies as an independent breast cancer risk factor^[17,18]^. In such instances, ultrasonography becomes a useful supplement, providing improved lesion characterization in dense breast and in the differentiation of solid and cystic masses. Additionally, ultrasonography is the imaging modality of first choice in patients aged <30 years and in pregnant and lactating women because of its safety and absence of ionizing radiation^[19,20]^.

The Breast Imaging Reporting and Data System (BI-RADS) offers a uniform lexicon to report breast imaging results, enhancing radiologist and clinician communication, and directing management plans^[21,22]^. Although ultrasonography and mammography have been used in conjunction in the evaluation of breast cancer, the comparative diagnostic accuracy of the two techniques continues to be the focus of research. Previous research has reported varied outcomes, which often depend on patient age, breast density, lesion type, and the operator’s skill level.

Histopathological examination remains the definitive diagnostic reference^[23,24]^. Therefore, evaluating imaging modalities against histopathology is essential for validating their reliability. This study seeks to assess the diagnostic performance of ultrasonography and mammography in detecting breast cancer, with histopathology serving as the reference standard, to guide optimal imaging practices in clinical settings. By establishing a clear understanding of the strengths and limitations of each imaging modality, this study contributes to advancing breast cancer diagnosis, improving patient care, and informed decision-making by healthcare professionals.

## Materials and methods

### Study design, setting, duration, and ethical considerations

A cross-sectional descriptive study was conducted at a tertiary care hospital, over a period of 9 months from 20 May 2024 to 10 January 2025. Written informed consent was taken from all the participants. All information and data collection were kept confidential. Ethical approval for this study was provided by the Institutional Review Board of the Research Ethics Committee (REC) on April 12, 2024. Our study has been reported in line with strengthening the reporting of cohort, cross-sectional, and case-control studies in surgery (STROCSS) guidelines^[25]^.

**HIGHLIGHTS**

- Breast cancer remains the most common malignancy in women, requiring accurate and timely diagnosis.
- Ultrasound showed consistent diagnostic accuracy of 86.3% in both breasts.
- Mammography was superior for detecting microcalcifications and subtle architectural distortions.
- Ultrasound had a strong negative predictive value (≥95.7%) and outperformed in dense breasts, reinforcing its value in premenopausal women.
- Combined use of ultrasound and mammography improved overall diagnostic reliability. Study supports dual-modality imaging to enhance early breast cancer detection.

### Inclusion and exclusion criteria

This study included female patients aged between 30 and 90 years who presented with clinically suspicious breast lesions and were referred for diagnostic imaging followed by histopathological confirmation. All participants were required to have undergone both mammography and ultrasonography within 6 months prior to biopsy. Only those lesions deemed most suspicious by imaging, in cases of multiple lesions, were selected for evaluation. Patients were included through a non-probability consecutive sampling technique, ensuring that all eligible participants during the study period were enrolled.

Patients were excluded if they had previously confirmed benign lesions, breast implants, or were unable to undergo either imaging or biopsy procedures. Additional exclusions included pregnant or lactating women, as well as those intending to become pregnant during the study period, due to potential contraindications to radiological procedures. Individuals with incomplete imaging or histopathology data were also excluded to ensure the integrity of diagnostic performance comparisons.

### Imaging protocol and evaluation

Ultrasound was done with a Toshiba Aplio system and a high-frequency linear probe (12–18 MHz). Both breasts and axillary tail were scanned radially, and patients were positioned supine with arms behind their heads and relaxed. Contralateral obliques were employed to examine lateral lesions and axilla. Mammography involved standard craniocaudal (CC) and mediolateral oblique (MLO) views. All images were assessed by an expert radiologist. The procedures of both modalities were done within 6 months of biopsy, and there was evaluation of only the most suspicious lesion of each breast.

### Data collection and statistical analysis

Data collection for this cross-sectional study was carried out over a 9-month period at the radiology department of a tertiary care hospital. All participants were female patients presenting with clinically suspected breast malignancy, who were referred for both mammography and ultrasonography before undergoing histopathological biopsy. Prior to imaging, informed consent was obtained, and ethical approval was secured. Each patient underwent a detailed breast imaging protocol including standard CC and MLO mammographic views, and bilateral ultrasound using a high-frequency linear probe (12–18 MHz) on a Toshiba Aplio system. The most suspicious lesion per breast was selected for evaluation in cases with multiple lesions. Imaging evaluations were conducted by an experienced radiologist blinded to the histopathological findings to reduce bias.

Following imaging, core needle biopsies or excisional biopsies were performed, and histopathological results were considered the gold standard for diagnosis. Imaging parameters such as lesion margins, echogenicity, location, and BI-RADS classification were recorded for ultrasonography, while mammography assessments included calcifications, architectural distortions, nipple retraction, skin thickening, and mammographic density.

The data were entered and analyzed using SPSS version 29.0. Descriptive statistics were used to summarize patient demographics and lesion characteristics. Categorical variables were expressed as frequencies and percentages. For the comparative diagnostic performance of mammography and ultrasonography, key metrics – sensitivity, specificity, positive predictive value (PPV), negative predictive value (NPV), and diagnostic accuracy – were calculated using 2 × 2 contingency tables against histopathological outcomes. The chi-square (χ^2^) test was applied to determine the statistical significance of associations between imaging findings and histopathological results, with a *P*-value of <0.05 considered statistically significant. Confidence intervals (95% CIs) were also computed for performance metrics to assess reliability.

## Results

Among the 240 female participants, 13.3% reported a family history of breast carcinoma. A majority (91.3%) were married, while 8.7% were unmarried. Menstrual status data showed that 60.8% were premenopausal, and 39.2% were postmenopausal (Table 1).

**Table 1:** Demographic characteristics of the study’s participants

Parameter		Frequency	Percentage (%)	Total
Family History	Yes	20	13.3%	240 (100%)
	No	220	86.7%	
Marital status	Married	219	91.3%	240 (100%)
	Unmarried	21	8.7%	
Menstrual Status	Premenstrual	146	60.8%	240 (100%)
	Postmenstrual	94	39.2%	

Ultrasound examination of the right breast revealed that 85.4% of lesions had smooth margins, while 14.6% had irregular margins. For the left breast, 93.8% of lesions were smooth-margined, and 6.2% were irregular. The upper outer quadrant (UOQ) was the most common lesion location in both right (75.4%) and left (75.8%) breasts. Echogenicity assessment showed that 67.9% of right breast lesions and 75.4% of left breast lesions were hyperechoic, whereas hypoechoic lesions were less common (32.1% in right and 24.6% in left breast; Table 2).

**Table 2:** Ultrasound and mammographic imaging characteristics of breast lesions

Breast ultrasound parameters	Frequency	Percentage	Total
Right breast margins			
Smooth	205	85.4%	240 (100%)
Irregular	35	14.6%	
Left breast margins			
Smooth	225	93.8%	240 (100%)
Irregular	15	6.2%	
Right breast lesion location			
UOQ	181	75.4%	240 (100%)
UIQ	20	8.3%	
LOQ	10	4.2%	
LIQ	13	5.4%	
Retroerolar	16	6.7%	
Left breast lesion location			
UOQ	182	75.8%	240 (100%)
UIQ	20	8.3%	
LOQ	13	5.4%	
LIQ	15	6.3%	
Retroerolar	8	3.3%	
Missing	2	0.80%	
Right breast echogenicity			
Hyperechoic	163	67.9%	240 (100%)
Hypoechoic	77	32.1%	
Left breast echogenicity			
Hyperechoic	181	75.4%	240 (100%)
Hypoechoic	59	24.6%	
**Mammographic parameters**	**Frequency**	**Percentage**	**Total**
Right breast calcifications			
No	192	80%	240 (100%)
Yes	48	20%	
Left breast calcifications			
No	205	85.4%	240 (100%)
Yes	35	14.6%	
Right breast perilesional architectural distortion			
No	217	90.4%	240 (100%)
Yes	23	9.6%	
Left breast perilesional architectural distortion			
No	225	93.7%	240 (100%)
Yes	15	6.3%	
Right breast nipple retraction			
No	232	96.7%	240 (100%)
Yes	8	3.3%	
Left breast nipple retraction			
No	238	99.2%	240 (100%)
Yes	2	0.8%	
Right breast skin thickening			
No	227	94.6%	240 (100%)
Yes	13	5.4%	
Left breast skin thickening			
No	228	95%	240 (100%)
Yes	12	5%	
Right breast mammographic echogenicity			
Hyperdens	66	30.8%	240 (100%)
Hypodens	174	69.2%	
Left breast mammographic echogenicity			
Hyperdens	56	23.3%	240 (100%)
Hypodens	184	76.7%	
Right breast BI-RADS			
1	84	35%	240 (100%)
2	59	24.6%	
3	51	21.3%	
4	31	12.9%	
5	12	5%	
6	3	1.3%	
Left breast BI-RADS			
1	122	50.8%	240 (100%)
2	44	18.3%	
3	38	15.8%	
4	28	11.7%	
5	3	1.3%	
6	5	2.1%	

Mammographic evaluation identified calcifications in 20% of lesions of the right and 14.6% of the left breast. Perilesional architectural distortion occurred in 9.6% and 6.3% of lesions of the right and left breasts, respectively. Nipple retraction was uncommon, occurring in 3.3% (right) and 0.8% (left). Thickening of skin occurred in 5.4% (right) and 5% (left). Echogenicity findings identified higher percentages of hypodense options in both breasts (69.2% right, 76.7% left). The BI-RADS assessment established that the majority of lesions were in categories I–IV, with BI-RADS I being the highest in both right (35%) and left (50.8%) breasts (Table 2).

The cross-tabulation between mammography and ultrasound diagnosis of the right breast can be depicted in Table 3. Of the total 240 cases, both modalities detected 67 cases as positive and both detected 148 cases as negative. Both detected discrepancies in 10 cases where mammography was positive and ultrasound was negative and in 15 cases where ultrasound was positive and mammography was negative. If ultrasound and mammography findings of the right breast were compared, ultrasound had a sensitivity of 81.7% and a specificity of 93.7%. The PPVs and NPVs were 87% and 90.8%, respectively. The overall accuracy of diagnosis was calculated to be 89.6%, and there was a statistically significant association between them (χ^2^ = 120.45, *P* < 0.001).

**Table 3:** Comparative cross-tabulation of ultrasound and mammography diagnoses: ultrasound vs. mammography diagnosis of right breast lesions

**Ultrasound vs. mammography**	**Present**	**Absent**	**Total**
Present	67 (27.9%)	10 (4.2%)	77 (32.1%)
Absent	15 (6.3%)	148 (61.7%)	163 (67.9%)
Total	82 (34.2%)	158 (65.8%)	240 (100)
**Performance metrics**			
Sensitivity	81.7% (95% CI: 71.4%–89.2%)		
Specificity	93.7% (95% CI: 88.5%–96.7%)		
Positive predictive value	87.0% (95% CI: 77.0%–93.2%)		
Negative predictive value	90.8% (95% CI: 85.3%–94.5%)		
Diagnostic accuracy	89.6% (95% CI: 84.9%–93.0%)		

In Table 4, cross-tabulation of left breast results, both ultrasound and mammography were positive in 44 cases and both were negative in 176 cases. In five cases, there was positive ultrasound and negative mammography, and in 15 cases, there was positive mammography and missed lesions by ultrasound. Left-sided comparison of results provided a higher sensitivity of 89.8% and a higher specificity of 97.2%. PPV was 89.8%, and NPV was 97.2%, reflecting strong diagnostic performance. The accuracy of diagnosis was 95%, and the chi-square test supported significant findings (χ^2^ = 114.61, *P* < 0.001).

**Table 4:** Comparative cross-tabulation of ultrasound and mammography diagnoses: ultrasound vs. mammography diagnosis of left breast lesions

Ultrasound vs. mammography	Present	Absent	Total
Present	44 (18.3%)	5 (2.1%)	49 (20.4%)
Absent	15 (6.3%)	176 (73.3%)	191 (79.6%)
Total	59 (24.6%)	181 (75.4%)	240 (100%)
**Performance metrics**			
Sensitivity	89.8% (95% CI: 76.2%–96.3%)		
Specificity	97.2% (95% CI: 93.7%–98.8%)		
Positive predictive value	89.8% (95% CI: 76.2%–96.3%)		
Negative predictive value	97.2% (95% CI: 93.7%–98.8%)		
Diagnostic accuracy	95.0% (95% CI: 91.4%–97.3%)		

Comparison with histopathology of the right breast revealed 48 cases as proved to be invasive and 34 as noninvasive out of mammography-positive results. On the other hand, 148 cases with mammography-negative results were indeed noninvasive, and there were missed cases of invasive lesions to the tune of 10, depicting an element of under-detection. Mammographic findings of the right breast compared to histopathology revealed a sensitivity of 82.8% and a specificity of 81.3%. The PPV was found to be 58.5%, and the NPV was observed to be 93.7%, with an accuracy of 81.7%. The results were statistically significant (χ^2^ = 56.38, *P* < 0.001; Table 5).

**Table 5:** Comparative cross-tabulation of mammography and histopathology: mammography vs. histopathology diagnosis of the right breast

**Mammography vs. histopathology**	**Invasive**	**Noninvasive**	**Total**
Present	48(20%)	34(14.2%)	82(34.2%)
Absent	10(4.2%)	148(61.7%)	158(65.4%)
Total	58(24.2%)	182(76.8%)	240(100%)
**Performance metrics**			
Sensitivity	82.8% (95% CI: 70.6%–90.9%)		
Specificity	81.3% (95% CI: 75.7%–87.0%)		
Positive predictive value	58.5% (95% CI: 46.9%–68.0%)		
Negative predictive value	93.7% (95% CI: 88.8%–96.6%)		
Diagnostic accuracy	81.7% (95% CI: 76.8%–86.6%)		

Among left breast cases, mammography identified all 36 of those that were later confirmed by histopathology to be invasive. Mammography identified 13 as false positives as well and reported them as noninvasive. No invasion was missed, and 191 noninvasive cases were identified as truly negative, reflecting a strong correlation with histopathological results. On the left side, mammography produced perfect sensitivity (100%), as well as high specificity (93.6%), with a PPV of 73.5% and an NPV of 100%. Diagnostic accuracy was 94.6%, reflecting its high reliability as an apparatus on the left side. Statistical testing was highly significant (χ^2^ = 109.21, *P* < 0.001; Table 6).

**Table 6:** Comparative cross-tabulation of mammography and histopathology: mammography vs. histopathology diagnosis of the left breast

Mammography vs. histopathology	Invasive	Noninvasive	Total
Present	36(15.0%)	13(5.4%)	49(20.4%)
Absent	0(0%)	191(79.6%)	191(79.6%)
Total	36(15.0%)	204(85%)	240(100%)
**Performance metrics**			
Sensitivity	100% (95% CI: 90.4%–100%)		
Specificity	93.6% (95% CI: 89.1%–96.3%)		
Positive predictive value	73.5% (95% CI: 58.3%–85.0%)		
Negative predictive value	100% (95% CI: 97.7%–100%)		
Diagnostic accuracy	94.6% (95% CI: 90.9%–97.2%)		

Right-sided ultrasound identified 51 invasive and 26 noninvasive cases among its positives. Among those labeled negative by ultrasound, 95 were truly noninvasive, but 7 invasive cases were missed. The crosstab shows a generally reliable detection pattern with minimal misclassification. Right-sided ultrasound compared to histopathology yielded a sensitivity of 87.9%, a specificity of 85.7%, a PPV of 66.2%, and an NPV of 95.7%. The overall diagnostic accuracy was 86.3%, and the findings were statistically significant (χ^2^ = 69.73, *P* < 0.001; Table 7).

**Table 7:** Diagnostic performance of ultrasound compared with histopathology: ultrasound vs. histopathology diagnosis of the right breast

Ultrasound vs. histopathology	Invasive	Noninvasive	Total
Present	51 (21.3%)	26 (10.8%)	77 (32.1%)
Absent	7 (2.9%)	156 (65%)	163 (67.9%)
Total	58 (24.2%)	182 (75.8%)	240 (100%)
**Performance metrics**			
Sensitivity	87.9% (95% CI: 76.7%–94.6%)		
Specificity	85.7% (95% CI: 79.6%–90.3%)		
Positive predictive value	66.2% (95% CI: 54.7%–75.9%)		
Negative predictive value	95.7% (95% CI: 91.2%–97.9%)		
Diagnostic accuracy	86.3% (95% CI: 81.3%–90.2%)		

Ultrasound identified 31 invasive and 28 noninvasive lesions on the left side of the breast. Five invasive cases were missed by ultrasound and revealed themselves solely by histopathology. Of ultrasound-negative cases, 176 were noninvasive, indicating predominantly correct differentiation between benign and malignant lesions. The specificity and sensitivity of ultrasound in the left breast were both around 86.3% and 86.1% respectively, with a PPV of 52.5% and a high NPV of 97.2%. The diagnostic accuracy was uniform at 86.3%, and the findings were significant at a statistical level (χ^2^ = 55.53, *P* < 0.001; Table 8).

**Table 8:** Diagnostic performance of ultrasound compared with histopathology: ultrasound vs. histopathology diagnosis of the left breast

Ultrasound vs. histopathology	Invasive	Noninvasive	Total
Present	31 (12.9%)	28 (11.7%)	59 (24.6%)
Absent	5 (2.1%)	176 (73.3%)	181 (75.4%)
Total	36 (15%)	204 (85%)	240 (100%)
**Performance metrics**			
Sensitivity	86.1% (95% CI: 70.5%–94.2%)		
Specificity	86.3% (95% CI: 80.5%–90.7%)		
Positive predictive value	52.5% (95% CI: 38.1%–66.6%)		
Negative predictive value	97.2% (95% CI: 93.4%–98.9%)		
Diagnostic accuracy	86.3% (95% CI: 81.2%–90.1%)		

Table 9 shows a detailed overview of ultrasonography and mammography diagnostic performance compared to histological findings in detecting breast carcinoma. Sensitivity, specificity, PPV, NPV, and total diagnostic accuracy are enumerated separately for the right and left breasts. These performance values represent each modality’s ability to identify both true positive and true negative cases correctly. The table also shows the high consistency of ultrasonography diagnosis on both breasts concerning NPV, which reflects its usefulness in excluding malignancy. Mammography was somewhat variable but had an excellent left-sided sensitivity and good specificity on both breasts. The combined evaluation of both sets of measurements provides a useful appraisal of each imaging modality’s clinical utility and informs optimal breast cancer evaluation diagnostic pathways.

**Table 9:** Summary of diagnostic performances

Comparison	Sensitivity	Specificity	PPV	NPV	Diagnostic accuracy	Remarks
Ultrasound vs. mammography (right)	81.7%	93.7%	87.0%	90.8%	89.6%	Good agreement; high NPV
Ultrasound vs. mammography (left)	89.8%	97.2%	89.8%	97.2%	95.0%	Excellent performance
Mammography vs. histopathology (right)	82.8%	81.3%	58.5%	93.7%	81.7%	Good sensitivity, low PPV
mammography vs. histopathology (left)	100%	93.6%	73.5%	100%	94.6%	Best overall metrics
ultrasound vs. histopathology (right)	87.9%	85.7%	66.2%	95.7%	86.3%	Strong NPV; few false negatives
Ultrasound vs. histopathology (left)	86.1%	86.3%	52.5%	97.2%	86.3%	Balanced metrics; high NPV

## Discussion

This study evaluated the diagnostic accuracies of both ultrasonography and mammography for identifying breast carcinoma, using histopathology as the definitive reference. The analysis revealed modality-specific weaknesses as well as strengths, which are consistent with previously reported findings. Ultra-sonography produced reliable diagnostic results, particularly in dense breasts, although mammography was more effective at detecting subtle microcalcifications and architectural distortions. These findings provide insight into how each imaging modality influences clinical decision-making and support the continued use of both techniques in complementary diagnostic pathways.

In this investigation, the use of ultrasonography revealed consistent accuracy (86.3%) in both breasts, highlighting its reliability in assessing dense breast tissue, often a limitation of mammo-graphy. The high rates of NPVs (95.96–97.27%) further confirm its utility in ruling out malignancies, especially among younger and premenopausal women with dense parenchyma. This is consistent with results of the ACRIN 6666 trial by Berg *et al*, where additional ultrasound increased cancer detection by 28% among women with dense breasts, pinpointing lesions undetected by mammography alone^[26]^. Kolb *et al* similarly stressed ultrasound’s advantage in dense breasts, with a sensitivity of 97% compared to that of mammography at 63% in heterogeneously dense tissue^[27]^. The prevalence of hyperechoic lesions (67.8–75.3%) and smooth borders (85.6–93.8%) in our population further supports previous findings by Lamb *et al*, who linked these sonographic features to benign or earlier-stage malignancies, supporting confidence in diagnosis^[28]^.

Mammography was highly variable, with accuracy rates of 81.7% in the right breast and 94.6% in the left. The high sensitivity (100%) and specificity (93.6%) of the left breast can be attributed to its anatomical position in imaging and to lesion features like calcification patterns. Interestingly, mammography was able to detect microcalcifications in 19.9% of lesions of the right breast and in 14.4% of those of the left breast, and detection of such microcalcifications is crucial due to their association with ductal carcinoma *in situ* (DCIS). This is corroborated by Shetty who emphasized mammography’s unrivaled ability to detect microcalcifications, an indication of malignancy in its earliest stages^[15]^. The lower sensitivity (82.8%) and PPV (58.5%) of mammography in the right breast can be attributed to technical or anatomical issues like tissue overlap or imperfect compression – a source of limitation as well as in studies by Brem *et al* who recommended the use of adjunct imaging in cases of mammographic indeterminacy^[29]^. The mammographic performance difference between breasts mirrors Skaane *et al*’s findings, where they noted corresponding asymmetry in digital mammography due to differences in breast density distribution and position^[30]^. Our study’s improved accuracy of the left breast mirrors Wang *et al*’s findings where mammography sensitivity was noted to increase to 92% following well-positioned examinations, calling for standardized procedures^[31]^.

BI-RADS categorization identified the majority of lesions as II–V, with higher specificity in categories 3 and 5 (100%), aligning with Jia *et al*, who documented BI-RADS 4A and 5 as the most sensitive categories (70.1–80.3%)^[32]^. The dominance of BI-RADS I (34.9–50.7%) in our group emphasizes the system’s ability to stratify low-risk lesions and prevent unnecessary biopsies. Burnside *et al* also stressed BI-RADS’s utility in standardizing reporting and managing, especially distinguishing between benign and suspicious calcifications^[22]^. Whereas ultrasonography dominated lesion characterization and dense breast assessment, mammography’s advantage in microcalcification and architectural distortion detection reiterates its utility. This duality is reflective of international consensus like that of the European Society of Breast Imaging (EUSOBI) guidelines supporting dual modalities in complete assessment by Mann *et al*^[18]^. An example here is in occult lesions detected by mammography, where US offers significant adjunctive utility, as illustrated by Madjar in increasing cancer detection by 20% when both modalities were employed^[19]^.

Two typical cases from our cohort that provide these diagnostic contrasts are represented. Case 1: A 34-year-old premenopausal woman presented with a palpable breast lump. No suspicious lesion could be traced on mammography due to the dense breast parenchyma, but ultrasonography detected a hypoechoic irregular mass, which later turned out to be invasive ductal carcinoma on histopathology. This case well delineates the advantage of ultrasonography in the evaluation of dense breasts and malignancies obscured on mammography. Case 2: A 57-year-old postmenopausal female was subjected to mammography, which revealed clustered microcalcifications very suspicious of DCIS. These could not be demonstrated by ultrasonography and therefore outlined the sensitivity of mammography in the detection of subtle microcalcifications and early non-palpable lesions. Together, these examples hint at the complementary nature of mammography and ultrasonography and thereby reinforce that their combined application can optimize diagnostic accuracy and early detection in diverse breast tissue types.

The implications of the study support individualized screening practices. In areas of high prevalence of breast density, like South Asia, breast ultrasound as the top priority, in combination with mammography, can prevent late-stage presentations. This is in agreement with Sood *et al*, whose meta-analysis of 46 studies supported ultrasound as an initial screening modality in low-resource environments with limited mammographic facilities. However, in populations with higher numbers of DCIS or calcifications, mammography continues to be crucial^[33]^. Moreover, advances in oncologic imaging have also been shown to enhance diagnostic precision and inform treatment strategies, further underscoring the clinical value of imaging-based evaluation across different cancer contexts^[34,35]^.

### Limitations and recommendations

This study has several limitations that should be acknowledged. First, it was conducted in a single tertiary care center, which may limit the generalizability of the findings to broader populations or diverse healthcare settings. Second, although the sample size was adequate for analysis, it may not fully represent variations in breast cancer presentation across different ethnic, socioeconomic, or geographic groups. Third, all imaging interpretations were performed by a single expert radiologist, which precludes assessment of interobserver variability and may have introduced interpretation bias. Fourth, the study excluded patients with previously confirmed benign lesions, breast implants, and pregnant and lactating women, which may restrict the applicability of the findings to these subgroups. Finally, while histopathology was used as the gold standard, the study did not account for molecular subtyping of breast cancer, which may influence imaging characteristics and diagnostic accuracy.

Future research should consider multi-center studies **with larger** and more diverse populations to improve the external validity of the findings. The inclusion of multiple radiologists with different levels of professional expertise is recommended for assessing the inter-observer variability using statistical metrics such as Cohen’s kappa, as indicated by Wang *et al*^[36]^ in increasing diagnostic reproducibility. Advanced imaging modalities such as digital breast tomosynthesis, contrast-enhanced mammography, and breast MRI should be incorporated in comparative analyses to provide a broader diagnostic spectrum. Moreover, studies involving subgroups such as pregnant women, lactating women, and those with breast implants are warranted to enhance clinical applicability. Integration of artificial intelligence–based image analysis could also be explored to improve sensitivity and reduce observer-related variability. Finally, longitudinal studies examining how imaging findings influence treatment outcomes and long-term survival would provide valuable insights into the clinical utility of combined imaging approaches.

## Conclusion

This study highlights the complementary diagnostic value of ultrasonography and mammography in the evaluation of breast carcinoma. Ultrasound demonstrated consistent diagnostic accuracy and strong NPV, making it particularly valuable in premenopausal women and those with dense breast tissue. Mammography, on the other hand, showed superiority in detecting microcalcifications and subtle architectural distortions, underscoring its indispensable role in identifying DCIS and early-stage malignancies.

The findings suggest that while each modality has its limitations, their combined use offers the most reliable diagnostic pathway, particularly in resource-limited settings where delayed presentation of breast cancer remains a major concern. For clinical practice, integrating both modalities into routine diagnostic protocols can enhance early detection, improve patient stratification, and ultimately reduce breast cancer-related morbidity and mortality.

In conclusion, ultrasonography and mammography should not be viewed as competing techniques but rather as complementary tools. Their judicious use, guided by patient age, breast density, and clinical presentation, can ensure more accurate diagnoses and better-informed treatment planning. Strengthening diagnostic strategies through combined imaging can play a pivotal role in advancing breast cancer care, especially in LMICs, where early detection remains the cornerstone of improved survival.

## Data Availability

The datasets used and/or analyzed during the current study are available from the corresponding author on reasonable request.

## Research registration unique identifying number (UIN)

Name of the registry: Thai Clinical Trials Registry. Unique identifying number or registration ID: TCTR202409901054. Hyperlink to your specific registration (must be publicly accessible and will be checked): https://www.thaiclinicaltrials.org.
